# Preclinical Studies of Chiauranib Show It Inhibits Transformed Follicular Lymphoma through the VEGFR2/ERK/STAT3 Signaling Pathway

**DOI:** 10.3390/ph16010015

**Published:** 2022-12-22

**Authors:** Yuanfang Tang, Mengya Zhong, Guangchao Pan, Jinshui Tan, Chendi Xie, Yuelong Jiang, Jingwei Yao, Weihang Shan, Jiaqi Lin, Jiewen Huang, Yating Liu, Zhifeng Li, Bing Xu, Jie Zha

**Affiliations:** 1 Department of Hematology, The First Affiliated Hospital of Xiamen University and Institute of Hematology, School of Medicine, Xiamen University, Xiamen 361003, China; 2 Key Laboratory of Xiamen for Diagnosis and Treatment of Hematological Malignancy, Xiamen 361003, China; 3 State Key Laboratory of Cellular Stress Biology, Innovation Center for Cell Biology, School of Life Sciences, Xiamen University, Xiamen 361002, China; 4 School of Pharmaceutical Sciences, Xiamen University, Xiamen 361002, China

**Keywords:** transformed follicular lymphoma (t-FL), chiauranib, VEGFR2, VEGFR2/ERK/STAT3 signaling pathway

## Abstract

Transformed follicular lymphoma (t-FL), for which there is no efficient treatment strategy, has a rapid progression, treatment resistance, and poor prognosis, which are the main reasons for FL treatment failure. In this study, we identified a promising therapeutic approach with chiauranib, a novel orally developed multitarget inhibitor targeting VEGFR/Aurora B/CSF-1R. We first determined the cytotoxicity of chiauranib in t-FL cell lines through CCK-8, EdU staining, flow cytometry, and transwell assays. We also determined the killing effect of chiauranib in a xenograft model. More importantly, we identified the underlying mechanism of chiauranib in t-FL tumorigenesis by immunofluorescence and Western blotting. Treatment with chiauranib significantly inhibited cell growth and migration, promoted apoptosis, induced cell cycle arrest in G2/M phase, and resulted in significant killing in vivo. Mechanistically, chiauranib suppresses the phosphorylation level of VEGFR2, which has an anti-t-FL effect by inhibiting the downstream MEK/ERK/STAT3 signaling cascade. In conclusion, chiauranib may be a potential therapy to treat t-FL, since it inhibits tumor growth and migration and induces apoptosis by altering the VEGFR2/ERK/STAT3 signaling pathway.

## 1. Introduction

As the second most prevalent non-Hodgkin lymphoma (NHL), follicular lymphoma (FL) occasionally undergoes histologic transformation (HT) into a more severe variant known as transformed follicular lymphoma (t-FL) [1,2]. Studies have shown that between 30% and 50% of t-FL patients have extremely aggressive diffuse large B-cell lymphoma (DLBCL) or Burkitt lymphoma (BL) [3,4]. Therefore, t-FL has evolved into the central focus of clinical therapy, since it is linked to rapid disease progression, treatment resistance, and poor prognosis of patients [2,4]. New emergent therapeutic approaches, including rituximab alone and in combination with the cyclophosphamide, vincristine, doxorubicin, and prednisolone (CHOP) regimen, have significantly altered the clinical outcomes of individuals with t-FL [5,6]. However, these novel tactics typically fail or lose their initial impact. Recent advancements in the realm of small molecule inhibitors have identified alternative therapeutic options, which may provide an opportunity for individuals with t-FL who have had unsuccessful therapies and dismal outcomes.

Chiauranib (CS2164) is a novel orally developed multitarget inhibitor that inhibits three main pathways of tumorigenesis, including the angiogenesis-associated kinase VEGFR (VEGFR1, VEGFR2, VEGFR3), the mitotic-associated kinase Aurora B, and the chronic inflammation-associated kinase CSF-1R [7]. Compared with combined regimen therapy, the effect of multitarget drugs on each target can produce a synergistic effect, and the best therapeutic effect is achieved [8]. Promising results have shown that chiauranib has a lethal effect on a variety of solid tumors, including non-small cell lung cancer, liver cancer, colorectal cancer, and lymphoma [9,10,11]. Additionally, phase I clinical trials have been launched to evaluate the antitumor activity of chiauranib against the most prevalent primary malignancies and showed minimal harmful side effects [12]. Our earlier research found that chiauranib blocks VEGFR2 to prevent the growth of acute myeloid leukemia (AML) [13], but the efficacy of chiauranib in treating t-FL is still unknown.

Here, for the first time, to the best of our knowledge, we identified the antitumor activity of chiauranib in t-FL. The results showed that chiauranib inhibited cell proliferation, increased apoptosis, and induced cell cycle arrest in t-FL cells. Moreover, chiauranib exhibited lethal effects in a xenograft model. Mechanistically, chiauranib inhibits the MEK/ERK/STAT3 signaling pathway by reducing the phosphorylation level of VEGFR2. These findings provide an in-depth research direction for the clinical treatment of t-FL.

## 2. Results

### 2.1. Chiauranib Exhibits Dose- and Time-Dependent Cytotoxicity in t-FL Cells

**Figure 1:**
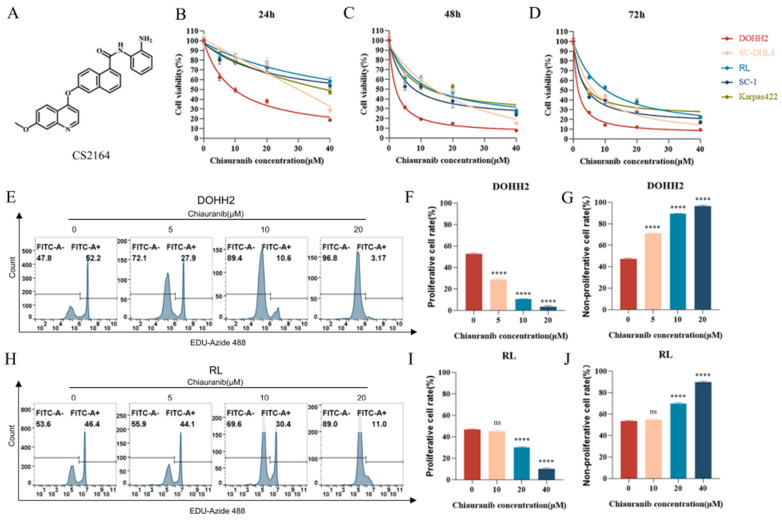
Chiauranib inhibited the proliferation of t-FL cells. (**A**) Chemical structure of the chiauranib. CS2164: N-(2-aminophenyl)-6-[(7-methoxy-4-quinolinyl)oxy]-1-naphthalene-carborxamide; Cell viability of t-FL cell lines including DOHH2, SU-DHL4, RL, SC-1, and Karpas422 tested by CCK8 assays after treatment with DMSO or chiauranib for (**B**) 24 h, (**C**) 48 h, or (**D**) 72 h. The results of EdU assays of (**E**) DOHH2 and (**H**) RL. Data from at least three independent experiments are shown in (**F**,**G**) DOHH2 and (**I**,**J**) RL (**** *p* < 0.0001).

First, we examined the ability of chiauranib to treat lymphoma in five t-FL cell lines: DOHH2, SU-DHL-4, RL, SC-1, and Karpas422. To investigate the antiproliferative impact, we performed a CCK-8 assay. Similar outcomes were achieved, with the proliferation rate of all t-FL cell lines strongly suppressed in a dose- and time-dependent manner after exposure to a series of prescribed doses of chiauranib (Figure 1A–C, Supplementary Figure S1). Each cell line’s IC_50_ was estimated precisely at 24 h, 48 h, or 72 h. As expected, the IC_50_ values at 72 h were lower than those at 48 h (Table 1).

EdU is a thymine nucleoside analog that can replace thymine (T) in replicating DNA molecules during cell proliferation. The intensity of fluorescently labeled EdU in cells can be used to measure cell proliferation [14]. The EdU experiment was used to examine changes in cell proliferation, and the findings revealed that EdU-positive cells, or cells that cease growing, gradually decreased as the concentration of chiauranib rose, whereas EdU-negative cells steadily increased (Figure 1D–I, Supplementary Figure S1). These results indicate that chiauranib reduces t-FL cell growth and that this reduction is dose- and time-dependent.

### 2.2. Chiauranib Induces Apoptosis in t-FL Cells

**Figure 2:**
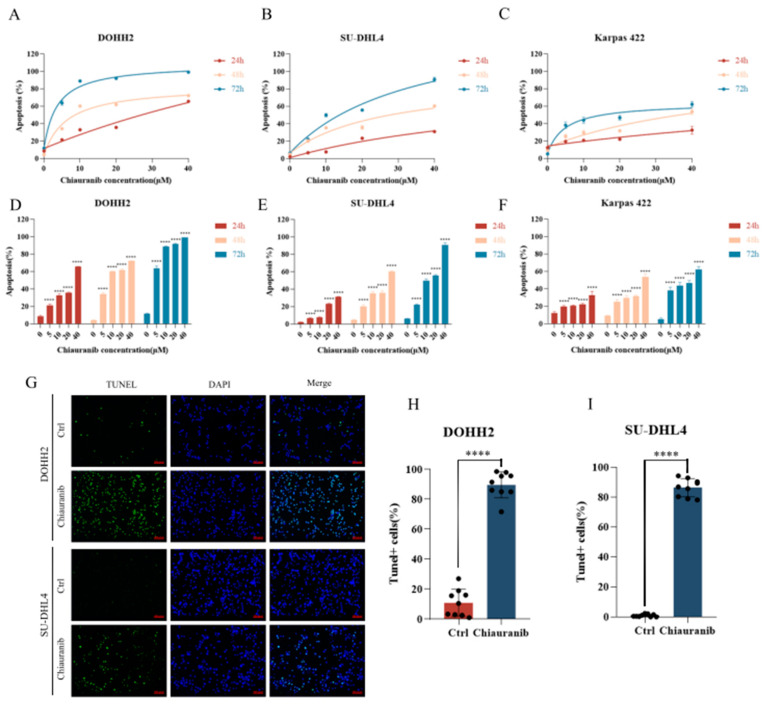
Chiauranib induced apoptosis of t-FL cells. The double staining of Annexin V and PI for cell apoptosis in (**A**) DOHH2, (**B**) SU-DHL4, and (**C**) Karpas422 cells. The statistical graph is shown for (**D**) DOHH2, (**E**) SU-DHL4, and (**F**) Karpas422. *p*-values represented by asterisks in the graphs represent statistically significant differences between each chiauranib concentration and vehicle control. (**G**) After treating with 40 µM chiauranib, the results of the TUNEL assay of DOHH2 and SU-DHL4 are photographed (proportional scale 1:100 pixel). Data from at least three independent experiments are shown for (**H**) DOHH2 and (**I**) SU-DHL4 (**** *p* < 0.0001).

Next, we examined the apoptotic status of t-FL cell lines after treatment. DOHH2, SU-DHL4, and Karpas422 cells were treated with different concentrations of chiauranib for 24, 48, and 72 h, and flow cytometry analysis with Annexin V/PI staining was performed to observe the rate of apoptosis. The experimental findings demonstrated that the apoptosis rate of DOHH2, SU-DHL4, and Karpas422 cells was dramatically increased with the extension of time and the rise in chiauranib concentration (Figure 2A–F). Additionally, we conducted TUNEL staining for additional confirmation, and the results showed that chiauranib significantly increased the number of apoptotic cells that were fluorescently labeled compared to that of the control group (Figure 2G–I). These findings indicate that chiauranib is capable of triggering apoptosis in t-FL cell lines, which could accumulatively contribute to its antilymphoma effect.

### 2.3. Chiauranib Causes G2/M Phase Cell Cycle Arrest

**Figure 3:**
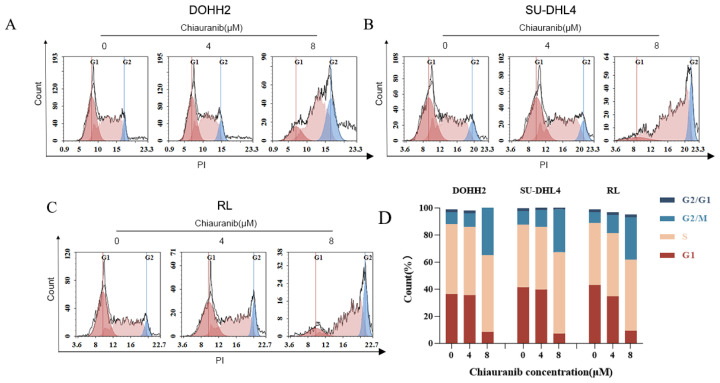
Chiauranib induces cell cycle arrest in the G2/M phase. The cell cycle of (**A**) DOHH2, (**B**) SU-DHL4, and (**C**) RL cells was arrested in the G2/M phase after treatment with chiauranib. (**D**) The corresponding cell cycle histograms were calculated.

To further delineate the impacts of chiauranib on t-FL cells, we carried out cell cycle assays to investigate the effect of growth arrest on the cell cycle. DOHH2, SU-DHL4, and RL cells were labeled with PI after being exposed to various doses of chiauranib for 24 h, and flow cytometry was used to identify periodic alterations in the cells. As a result, the proportion of cells in the G2/M phase in the treated group was higher than that in the untreated control group (Figure 3). In contrast, the ratio of G0/G1 phase cells was significantly decreased, with the S phase cells showing no change. Given these findings, we conclude that chiauranib causes cell cycle arrest in the G2/M phase.

### 2.4. Chiauranib Inhibits the Phosphorylation of the VEGFR2/MEK/ERK/STAT3 Signaling Pathway in t-FL Cells

**Figure 4:**
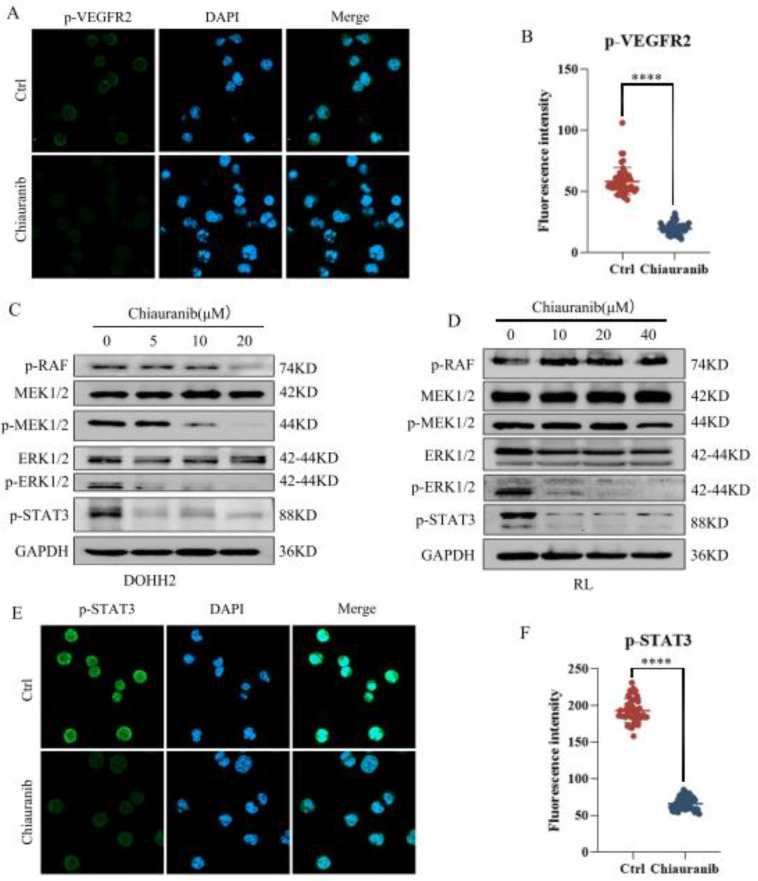
Chiauranib inhibits the VEGFR2/MEK/ERK/STAT3 signaling pathway. (**A**) Immunofluorescence shows that chiauranib administration (40 µM) for 24 h alters the phosphorylation levels of VEGFR2 in DOHH2 cells (proportional scale 1:100 pixel). (**B**) Data from at least three independent experiments. Western blotting assay to detect protein expression after chiauranib treatment in (**C**) DOHH2 and (**D**) RL cells for 24 h. After treatment with chiauranib (40 µM) for 24 h, the changes in the phosphorylation levels of STAT3 were examined by (**E**) immunofluorescence (proportional scale 1:100 pixel) and (**F**) data analysis (**** *p* < 0.0001).

In our earlier studies, the drug chiauranib, which targets lymphoma cells, was able to lower the levels of VEGFR2 phosphorylation [13]. We investigated the function of chiauranib in t-FL cells in terms of its molecular mechanism of action, and by using immunofluorescence, we discovered that it suppresses the phosphorylation of VEGFR2 in DOHH2 cells (Figure 4A,B). Research has shown that VEGFR2 regulates the phosphorylation level of downstream RAF by GRB2, which phosphorylates the target protein MEK1/2. ERK1/2 is a serine-threonine kinase activated by MEK1/2 phosphorylation [15]. We next performed a Western blot to determine which protein levels changed upon chiauranib treatment. As shown in Figure 4, chiauranib markedly diminished the phosphorylation levels of proteins downstream of VEGFR2, including p-RAF, p-MEK1/2, and p-ERK1/2, in a dose-dependent manner. Moreover, Western blotting and immunofluorescence revealed a reduction in the phosphorylation of STAT3 serine 727 (Figure 4), which is a target protein of ERK1/2 [16,17]. These results suggest that suppressing the VEGFR2/MEK/ERK/STAT3 signaling pathway allowed chiauranib to kill t-FL cells.

### 2.5. Chiauranib Alters the Levels of STAT3-Downstream Genes and Suppresses Angiogenesis in t-FL

**Figure 5:**
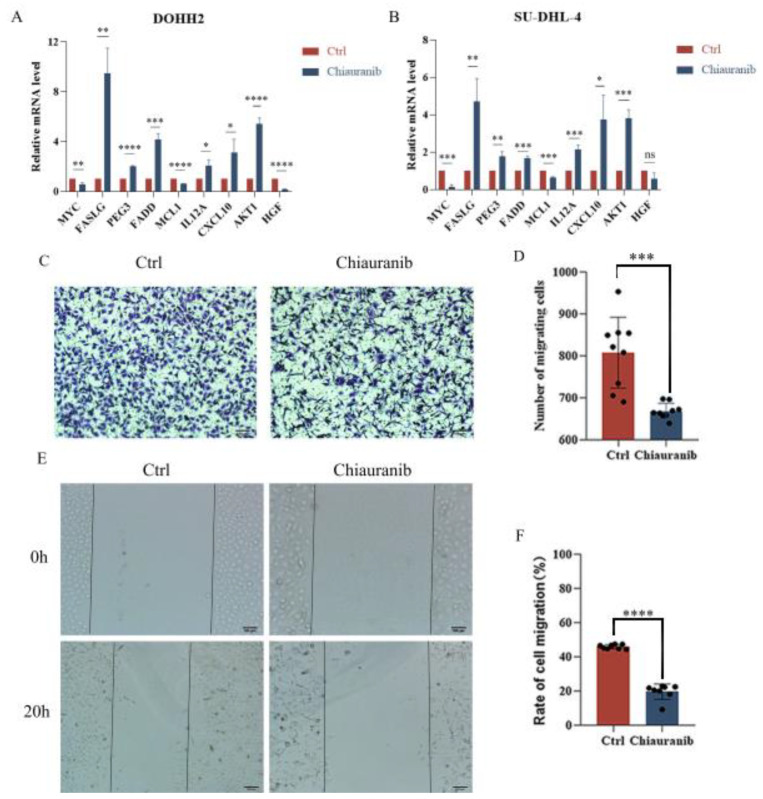
Chiauranib alters gene expression levels downstream of STAT3. Chiauranib (20 µM) inhibited the mRNA levels of downstream genes of STAT3, of (**A**) DOHH2, and (**B**) SU-DHL4 cells treated with chiauranib. (**C**,**D**) Transwell assay and (**E**,**F**) wound healing test to validate the migration of HUVECs following drug treatment (5 µM chiauranib; proportional scale 1:100 μm). (* *p* < 0.05;** *p* < 0.01;*** *p* < 0.001;**** *p* < 0.0001).

Numerous significant genes, including those involved in angiogenesis, cell migration, and cell survival, are regulated by STAT3 [18,19]. We used RT-PCR to show that the STAT3-downstream genes were altered to varying degrees by the action of chiauranib (Figure 5A,B). *FASLG*, *PEG3*, and *FADD* control cell death, while *MYC* controls cell growth, which suggests that cell viability is decreased after chiauranib treatment. *AKT1* and *HGF*, two angiogenic genes, were downregulated, whereas *IL12A* and *CXCL10*, as antiangiogenic genes, were elevated. This finding implies that chiauranib has the ability to prevent angiogenesis. We carried out cell migration studies, since the ability of vascular endothelial cells to move is crucial for angiogenesis. We found that following treatment with chiauranib, the capacity of HUVECs to migrate was considerably reduced (Figure 5C–F). All of these experimental findings demonstrated that chiauranib inhibits the angiogenesis of t-FL and further exhibits an antitumor effect.

### 2.6. In Vivo Efficacy of Chiauranib against t-FL Xenograft Mouse Models

**Figure 6:**
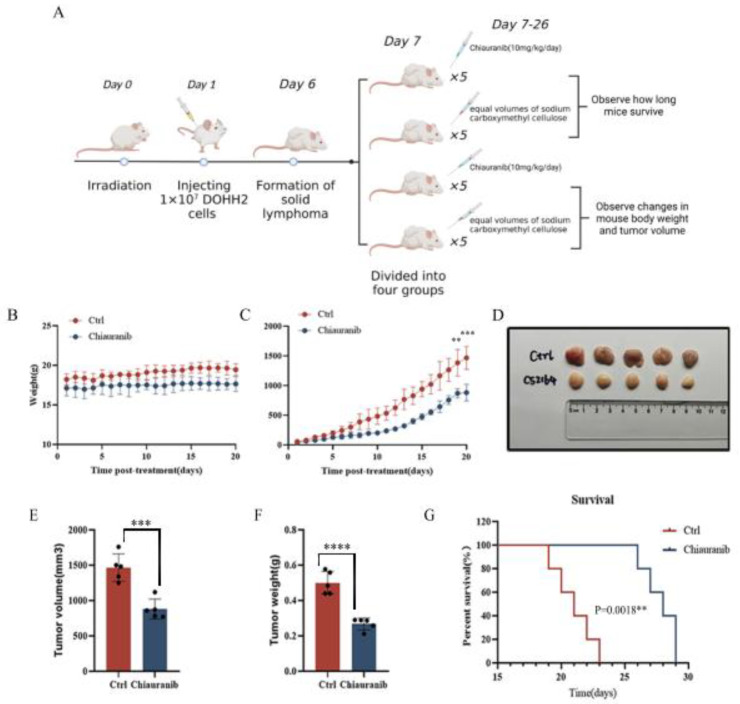
Chiauranib inhibits t-FL progression in xenograft models. (**A**) Schematic outline of the xenograft models. (**B**) Changes in body weight in mice. (**C**) The chiauranib-treated mice had lower tumor volumes than the control mice. (**D**) Images of tumors from xenograft models (n = 5). The chiauranib-treated mice also had lower (**E**) tumor volumes, (**F**) weights, and (**G**) improved survival curves. A total of 10 mice (5 control, 5 chiauranib) were monitored for survival for an additional ten days. (** *p* < 0.01, *** *p* < 0.001, **** *p* < 0.0001).

Finally, to assess the potential in vivo efficacy of chiauranib, we constructed t-FL xenograft models by subcutaneously injecting DOHH2 cells (1 × 10^7^) into NOD/SCID mice. As expected, the volume of lymphoma reached 100 mm^3^, and the mice were randomly divided into two groups. One group of mice was orally administered chiauranib (10 mg/kg/day), and the other group was given equal volumes of vehicle (Figure 6A). The mice in the chiauranib-administered group did not noticeably lose weight after 20 days of continuous treatment compared to those in the control group, showing that the mice maintained well-being during the chiauranib treatment (Figure 6B). Compared to the vehicle treatment, the inoculation treatment resulted in a significant reduction in the weight and volume of the tumor (Figure 6C–F). Through the image, chiauranib medication dramatically inhibited the development of tumors in DOHH2-bearing xenograft models (Figure 6D). Of note, chiauranib significantly prolonged survival in the chiauranib group (Figure 6G).

**Figure 7:**
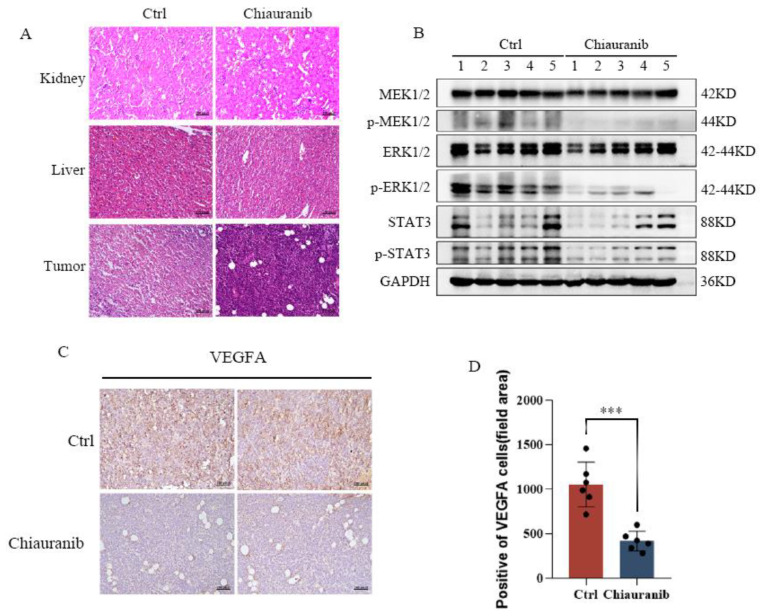
Chiauranib inhibits the MEK/ERK/STAT3 signaling pathway in vivo. (**A**) HE staining of the kidney, liver, and tumor (proportional scale 1:100 pixel). (**B**) Western blotting detection of the indicated protein extracted from tumor tissue (mice were orally administered 10 mg/kg/day chiauranib). (**C**) Immunohistochemistry assay of VEGFA in the chiauranib-treated (10 mg/kg/day) tumors compared with the control tumors (proportional scale 1:100 pixel). (**D**) Data from at least three independent experiments (*** *p* < 0.001).

For further tumor detection, we collected tumor tissues. HE staining of the mouse liver and kidney also demonstrates that chiauranib is less hazardous, and tumor tissues taken from the chiauranib-administered group displayed obvious nuclear shrinkage (Figure 7A). We found that the phosphorylation levels of MEK1/2, ERK1/2, and STAT3 were significantly reduced in the chiauranib group (Figure 7B). This finding is consistent with the results of our previous experiments (Figure 4). We used immunohistochemistry to confirm the change in *VEGFA*, and the results revealed that the group receiving chiauranib had considerably lower levels of *VEGFA* expression (Figure 7C,D). Together, these results show that chiauranib is capable of inhibiting tumorigenesis of t-FL by blocking the VEGFR2/MEK/ERK/STAT3 signaling pathway.

## 3. Discussion

Angiogenesis is essential for the growth and dissemination of malignancy [20]. High expression or elevated serum levels of vascular endothelial growth factor (VEGF), which results in increased microvessel density, are linked to metastatic formation and aggressive clinical prognosis, particularly in the lymphoma microenvironment [11,21]. Although the VEGF signaling pathway is thought to be a viable target for preventing the spread of a tumor, studies have shown that blocking or activating a certain specific target has little effect in the early phases of treatment and is insufficient for complicated disease [22,23]. Multitarget drug treatment is a fresh concept that systems biology has recently suggested for the ongoing development of pharmaceuticals [8]. Our previous study found that chiauranib, as a multitarget inhibitor of VEGFR/Aurora B/CSF-1R, exerted desirable antitumor effects in both AML and NHL [11,13]. The latest clinical trials have also confirmed its application prospects in a variety of tumors [12]. Considering the current disease progression and poor prognosis of t-FL patients, we investigated the antitumor impact of chiauranib in t-FL for the first time, providing a novel option for the treatment of t-FL patients.

Given the complexities of genetic alterations in t-FL, a universal treatment plan might not be effective for all patients [2,4]. In our initial experiments, we demonstrated that chiauranib has anticancer effects on five t-FL cell lines, DOHH2, RL, Karpas422, SU-DHL-4, and SC-1 cells, and presented substantial dose- and time-dependent effects. Additionally, chiauranib administration caused a much higher percentage of apoptosis, cell cycle arrest in the G2/M phase, and inhibition of the capacity to migrate, indicating that chiauranib suppresses t-FL by affecting several crucial pathways for t-FL development and cell proliferation. Notably, chiauranib effectively inhibited tumor development in an animal xenograft model without showing any lethal toxicity. Collectively, our data imply that t-FL therapy can be included in the list of conditions for which chiauranib is indicated.

As a key component of VEGF-induced signaling in vascular endothelial cells, VEGFR2 plays a role in both hematopoiesis and tumor angiogenesis [24,25]. The study found that chiauranib binds to the ATP pocket of VEGFR2 and inhibits the kinase activity, and the inhibition of capillary angiogenesis coincides with the phosphorylation of VEGFR2, thereby affecting tumor angiogenesis [7]. Additionally, VEGFR2 is expressed on lymphoma cells, and in NHL, VEGF receptors activate lymphoma cell autocrine signaling to promote tumor neovascularization [26]. Here, we discovered that chiauranib reduced the phosphorylation levels of VEGFR2 in t-FL cells as well as its downstream proteins, including RAF, MEK1/2, ERK1/2, and STAT3.

**Figure 8:**
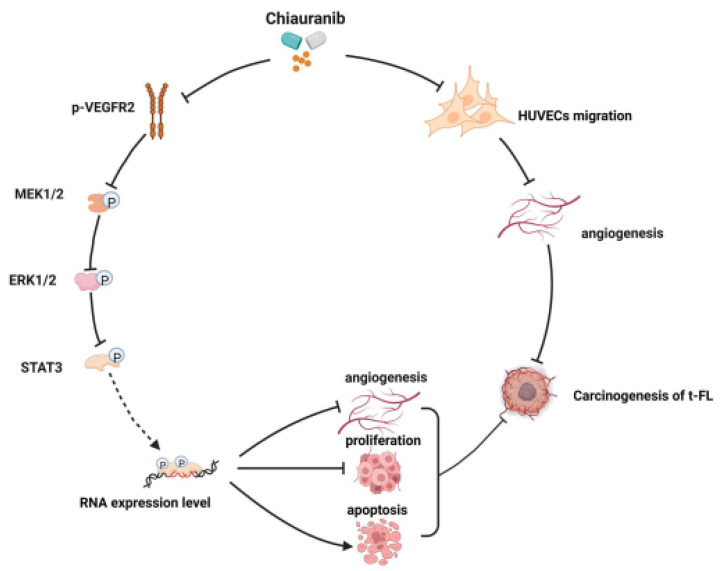
Schematic diagram of the mechanism by which chiauranib acts on t-FL.

Studies have shown that ERK1/2 directly phosphorylates STAT3 Ser727, and the phosphorylation of this site contributes to the activation of STAT3, which forms a dimer in the nucleus to function as a transcription factor [16,17]. Our findings showed that chiauranib significantly lowers the phosphorylation of STAT3 at Ser727, suggesting that chiauranib inhibits VEGFR2 and reduces the phosphorylation of ERK1/2, reducing STAT3 dimerization in the nucleus [18,19]. We also clarified that chiauranib influences the expression of downstream genes of STAT3 involved in tumorigenesis, such as inhibiting the angiogenesis-related genes *AKT1* and *HGF*, as well as increasing the expression of the proapoptotic genes *FASLG*, *PEG3*, and *FADD*. Likewise, our findings demonstrate that chiauranib therapy dramatically reduces the ability of HUVECs to migrate while considerably increasing the expression of the antiangiogenic genes *IL12A* and *CXCL10*. Thus, the mechanism of chiauranib-induced t-FL cytotoxicity is tightly connected to the VEGFR2/ERK/STAT3 signaling pathway (Figure 8).

In conclusion, chiauranib, a multitarget inhibitor, has the potential to be applied in t-FL as an effective anticancer therapy by potently inhibiting t-FL cell growth both in vitro and in vivo. Future research will focus further on determining whether other targets of chiauranib contribute to a synergistic lethal impact in t-FL, and follow-up clinical trials in t-FL patients will confirm its safety and efficacy.

## 4. Materials and Methods

### 4.1. Cell Culture and Chemical Reagents

The t-FL human cell lines DOHH2, RL, Karpas422, SU-DHL-4, and SC-1 were acquired from the Institute of Hematology at Xiamen University School of Medicine (Xiamen, Fujian, China), and they were grown in RPMI-1640 (Gibco, Carlsbad, CA, USA) with 10% fetal bovine serum (FBS, Gibco), 100 U/mL penicillin, and 100 g/mL (Invitrogen, Carlsbad, CA, USA). Human umbilical vein endothelial cells (HUVECs) were purchased from Guangzhou Cellcook Biotech Co., Ltd., (Guangzhou, Guangdong, China) and grown in endothelial cell growth medium (ECM, ScienCell, CA, USA) with 5% FBS, endothelial cell growth supplement (ECGS), and 100 U/mL antibiotic solution (ScienCell). All cell lines were cultured in a 37 °C incubator with a 5% CO_2_ environment.

Chiauranib (CS2164) was provided by Chipscreen Biosciences Co., Ltd. (Shenzhen, Guangdong, China) and dissolved in dimethyl sulfoxide (DMSO, Sigma, St. Louis, MO, USA) to create a stock solution. The chemical structures and full names of chiauranib are shown in Figure 1A. Before further usage, the stock solution (10 mM) was diluted using a culture medium to generate the concentrations and kept at −20 °C.

### 4.2. Cell Viability

A Cell Counting Kit-8 assay (CCK-8, Zeta Life, Menlo Park, CA, USA) was used to measure cell viability at various time intervals. Briefly, 100 μL of complete medium with chiauranib at the relevant doses was used to inoculate 2 × 10^5^ t-FL cells (DOHH2, RL, Karpas422, SU-DHL4, and SC-1) in a 96-well plate. IC50 values were determined by GraphPad Prism 8 (GraphPad Software, San Diego, CA, USA). The methodology used to determine the viability rate was as follows: viability rate (%) = OD value of (experimental group/control group) × 100%.

### 4.3. Cell Apoptosis and Cycle Arrest

DOHH2, Karpas422, and SU-DHL4 cells (2 × 10^5^ cells per well) were exposed to chiauranib at varying doses for 24, 48, and 72 h. Following the manufacturer’s instructions, the cells were washed with PBS before being treated with the Annexin V/PI Staining Kit (BD Biosciences, 556463, East Rutherford, NJ, USA). For the cell cycle, t-FL cells (5 × 10^5^ cells) were inoculated in a 12-well plate and exposed to various concentrations of chiauranib (0, 4, and 8 μM) for 24 h. The cells were then fixed in 70% ethanol overnight at 4 °C, resuspended in PBS containing RNase, and labeled with propidium iodide (PI) for 30 min in the dark (BD Pharmingen). Cell apoptosis and cell cycle distribution were determined by flow cytometry (Quanteon, ACEA Biosciences, San Diego, CA, USA). GraphPad Prism 8 and FlowJo software (San Carlos, CA, USA) were used for data analysis. Data from three independent triplicate experiments and the results are presented as the mean ± SD.

### 4.4. EdU Cell Proliferation Staining

DOHH2 and RL cells were treated with DMSO or chiauranib (20 μM) for 24 h and analyzed with a BeyoClick™ EdU Cell Proliferation Kit (Beyotime, Shanghai, China). Briefly, cells were incubated with 10 mM 5-ethynyl-2′ deoxyuracil nucleoside (EdU) for 2 h, permeated with 4% PFA for 15 min, and fixed for an additional 15 min with 0.3% Triton X-100 in PBS. The Click Reaction Mixture was combined with the cells for 30 min in complete darkness. Finally, flow cytometry revealed the presence of cells (Quanteon). Data analysis was performed with FlowJo software and GraphPad Prism 8, and the results are presented as the mean ± SD.

### 4.5. Wound Healing Assay

First, HUVECs were given a 24 h treatment with or without chiauranib (5 μM). Then, they were expanded to monolayer confluence on a 6-well plate of cultivated cells (2 × 10^6^ cells per well). The cells were scratched using a tip and washed three times with PBS. HUVECs were grown in complete ECM medium after the debris was cleared. Images were taken at 0 and 24 h using a Leica DM4B microscope after an additional 24 h of incubation (Leica, Wetzlar, Germany).

### 4.6. Transwell Assay

Matrigel-coated transwell plates were used to examine the capacity of HUVECs to migrate (Corning, Tewksbury, MA, USA). For 24 h, cells were either treated with or without chiauranib (5 μM). The next day, the treated HUVECs were transferred to transwell chambers containing serum-free ECM medium and to the bottom chamber containing complete ECM medium. After 24 h of incubation at 37 °C, the chambers were permeated with 4% paraformaldehyde (PFA) and stained with crystal violet staining solution (Beyotime). The cells on the top surface of the membrane eventually moved to the bottom surface, where they were counted and photographed under a microscope.

### 4.7. Western Blotting

Prior to Western blot analysis, chiauranib was administered at the appropriate concentrations to DOHH2 and RL cells for 24 h. Cells were collected and lysed using RIPA buffer (Beyotime, P0013C) after treatment. The following primary antibodies were used: VEGFR2 (A11127, 1:1000, Abclonal, Wuhan, China), P-VEGFR2 (AP0382, 1:1000, Abclonal), P-RAF (9421S, 1:1000, CST), MEK1/2 (A4868, 1:1000, Abclonal), P-MEK1/2 (AP0209, 1:1000, Abclonal), ERK1/2 (A4782, 1:1000, Abclonal), P-ERK1/2 (AP0974, 1:1000, Abclonal), STAT3 (A19566, 1:1000, Abclonal), P-STAT3 (AP0715, 1:1000, Abclonal), and GAPDH (AC001, 1:1000, Abclonal).

### 4.8. Quantitative RT-PCR

A total of 8 × 10^6^ cells were divided into two discs of 4 × 10^6^ cells each, with one group receiving chiauranib treatment and the other receiving DMSO treatment as a control. After 24 h, RNA was extracted using a SteadyPure Universal RNA Extraction Kit (AG21017, Accurate Biology, Changsha, China). Then the RNA was reverse-transcripted into cDNA with the use of the Evo M-MLV RT Master Mix (Accurate Biology). Following chiauranib therapy, we performed quantitative RT-PCR to identify genetic alterations in the *FASLG*, *PEG3*, *FADD*, *MYC*, *AKT1*, *HGF*, *IL12A*, and *CXCL10* genes. *ACTB* was used as an endogenous control. The primers used for quantitative RT-PCR are listed in Supplementary Table S1. The SYBR Green Premix Pro Taq HS qPCR Kit is the tool we used in RT-PCR (Accurate Biology). The 2^−ΔΔCT^ approach was employed to analyze relative gene expression.

### 4.9. Immunofluorescence

Slides were incubated with rat tail collagen overnight the day before the experiment. t-FL cells were added to glass slides and treated with chiauranib for 24 h. Then, cells were fixed with 4% PFA and then incubated in 5% BSA for one hour. Following overnight incubation at 4 °C with primary antibodies against P-VEGFR2 (AP0382, 1:200, Abclonal) and P-STAT3 (AP0715, 1:200, Abclonal), the cells were washed with PBST and then treated with a secondary antibody for 1 h before being stained with DAPI for 10 min.

DOHH2 and RL cells were exposed to chiauranib (20 μM) or left untreated for 24 h before TUNEL labeling. Next, using a One Step TUNEL Apoptosis Assay Kit (Beyotime) as directed by the manufacturer, the cells were handled. With 488 nm excitation, the positive cells glow green when observed under a fluorescence microscope.

### 4.10. Tumor Xenograft Models

The NOD-Prkdc^−/−^ IL2rg^−/−^ (NOD/SCID) mice (6 weeks old) utilized in this research were acquired from Xiamen University Animal Care and kept in a pathogen-free setting. The Animal Care and Use Committee and Ethics Committee of Xiamen University approved all animal investigations. After receiving 1 Gy of sublethal radiation, DOHH2 cells (1 × 10^7^) were subcutaneously injected into mice on the back. After the size of the subcutaneous tumors reached 100 mm^3^, mice were randomly split into four groups (n = 5). Two of the groups were mice given chiauranib orally at a dose of 10 mg/kg per day for 20 days. Mice from the other two groups received the vehicle as control groups during the same period. The weight change and tumor change were also noted every day. On day 20, one group of administered mice and one group of control mice were sacrificed for HE staining and immunohistochemistry (IHC) investigations (n = 5). The remaining two groups of mice (n = 5) continued to be observed for ten days to monitor for survival. On paraffin-embedded graft tissues, we labeled VEGFA (A0280, 1:200, Abclonal) using an immunohistochemical approach and then utilized an optical microscope to examine each slice (Nikon Eclipse Ci, Nikon).

### 4.11. Statistical Analysis

The data were examined statistically by utilizing GraphPad Prism 8 software. The mean ± SD was calculated using Student’s *t*-test or one-way analysis of variance (ANOVA). Values of *p* < 0.05 were regarded as statistically significant after three separate experiments were performed for each time point.

### 4.12. Ethics Approval and Consent to Participate

All animal experiments have been supervised and approved by the Laboratory Animal Ethics and Management Committee of Xiamen University.

## 5. Conclusions

By blocking the VEGFR2/ERK/STAT3 signaling cascade, this study recommends the novel multitarget inhibitor chiauranib as a potent anticancer treatment for transformed follicular lymphoma.

## Figures and Tables

**Table 1:** The IC_50_ values of chiauranib in t-FL cell lines.

Cell Lines	IC_50_ (μmol/L)		
	24 h	48 h	72 h
DOHH2	9.57 ± 1.27	1.75 ± 0.24	1.06 ± 0.19
SU-DHL4	28.72 ± 2.62	12.85 ± 0.51	5.84 ± 0.44
RL	68.19 ± 8.94	13.89 ± 2.13	10.37 ± 0.61
SC-1	61.33 ± 9.08	8.03 ± 2.05	3.68 ± 0.40
Karpas422	42.63 ± 5.14	13.31 ± 2.99	4.26 ± 1.22

## Data Availability

The data presented in this study are available on request from the corresponding author.

## Supplementary Materials

The following supporting information can be downloaded at: https://www.mdpi.com/article/10.3390/ph16010015/s1, Supplementary Table S1. The primers used for quantitative RT-PCR. Supplementary Figure S1. Chiauranib suppresses the growth of t-FL cell lines. The data of CCK-8 (mean ± S.D.) from at least three independent experiments are shown for (A) 24 h, (B) 48 h, or (C) 72 h. (D) The IC50 values of chiauranib-treated t-FL cells. (E) The results of EdU assay of SU-DHL4. (F,G) Data (mean ± S.D.) from at least three independent experiments are shown in SU-DHL4. (** *p* < 0.01; **** *p* < 0.0001).
