# Supplementary material for: Preclinical Studies of Chiauranib Show It Inhibits Transformed Follicular Lymphoma through the VEGFR2/ERK/STAT3 Signaling Pathway
Source: Pharmaceuticals (Basel). 2022 Dec 22;16(1):15. doi: 10.3390/ph16010015 (PMC9865968; doi:10.3390/ph16010015)
Supplement: Supplementary file 1 [file pharmaceuticals-16-00015-s001.zip › pharmaceuticals-2067467-supplementary.pdf]

Supplementary Table S1. The primers used for quantitative RT–PCR

Gene symbols	Sequence	
MYC	Forward	5'- TCCAGCTTGTACCTGCAGGATCTGA-3'
	Reverse	5'- CCTCCAGCAGAAGGTGATCCAGACT-3'
FASLG	Forward	5'- TTGGAGAAGCAAATAGGCCACC-3'
	Reverse	5'- AAGATTGAACACTGCCCCCA-3'
PEG3	Forward	5'- CTCACAACACAATCCAGGAC-3'
	Reverse	5'- TAGACCTCGACTGGTGCTTG-3'
FADD	Forward	5'- GCCATGGACCCGTTCCTGG-3'
	Reverse	5'- CTCGATGCTGTCGATCTTG-3'
MCL1	Forward	5'- CTTCGGAAACTGGACATCAA-3'
	Reverse	5'- GAAGAACTCCACAAACCCATC-3'
IL12A	Forward	5'- CACAAAGGAGGCGAGGTTCT-3'
	Reverse	5'- GGGTCAGGTTTGATGATGTCC-3'
CXCL10	Forward	5'- AGCAGTTAGCAAGGAAAGGTCT-3'
	Reverse	5'- GGAGGATGGCAGTGGAAGTC-3'
HGF	Forward	5'- CAGCTTTTTGCCTTCGAGCTA-3'
	Reverse	5'- TCTCGTAGGTCCTTGCACTT-3'
AKT1	Forward	5'- AAGACGGGAGCAGGCGG-3'
	Reverse	5'- GGTCTTGATGTACTCCCCTCG-3'
ACTB	Forward	5'- GCTGTGCTATCCCTGTACGC-3'
	Reverse	5'- TGCCTCAGGGCAGCGGAACC-3'

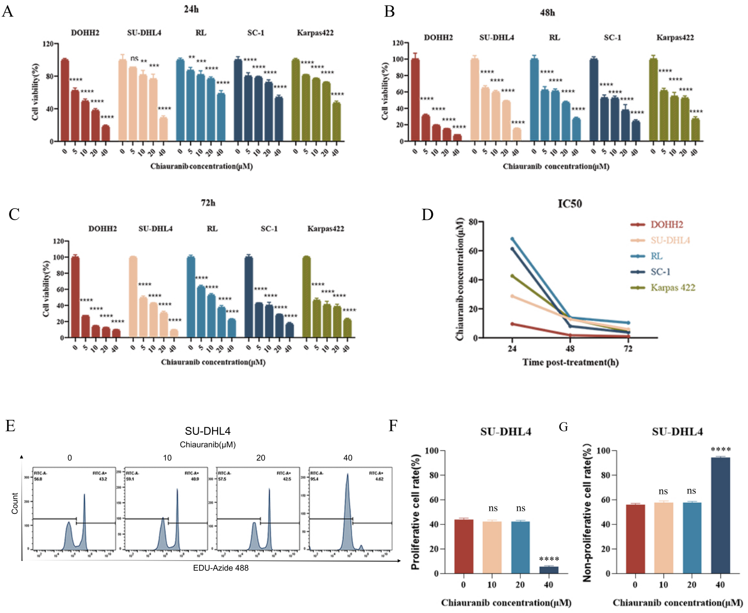

Supplementary Figure S1
